# Genome-Wide Survey of Donor Chromosomal Genes Involved in Trans-Kingdom Conjugation via the RP4-T4SS Machinery

**DOI:** 10.3390/microorganisms13030488

**Published:** 2025-02-22

**Authors:** Kazuki Moriguchi, Kazuyuki Nakamura, Yudai Takahashi, Kyoko Higo-Moriguchi, Kazuya Kiyokawa, Katsunori Suzuki

**Affiliations:** 1 Program of Basic Biology, Graduate School of Integrated Sciences for Life, Hiroshima University, Higashi-Hiroshima 739-8526, Japan; m233026@hiroshima-u.ac.jp (K.N.); kkiyokaw@hiroshima-u.ac.jp (K.K.); ksuzuki@hiroshima-u.ac.jp (K.S.); 2 Department of Biological Science, Faculty of Science, Hiroshima University, Higashi-Hiroshima 739-8526, Japan; 3 Fujita Health University School of Medicine, Toyoake 470-1192, Japan; khigo@fujita-hu.ac.jp

**Keywords:** genome-wide screening, IncP1-type plasmid, trans-kingdom conjugation, type IV secretion system, horizontal gene transfer

## Abstract

Trans-kingdom conjugation (TKC)/inter-domain conjugation is a horizontal gene transfer phenomenon that transfers DNA from eubacteria to eukaryotes and archaebacteria via a type IV secretion system encoded in IncP1-type broad-host-range plasmids. Although TKC is considered a potential gene introduction tool, donor chromosomal genes that influence TKC efficiency have rarely been analyzed, hindering targeted donor breeding. To identify potential TKC-related genes on a donor chromosome, a genome-wide screening of TKC-deficient mutants was performed using a comprehensive collection of *Escherichia coli* gene knockout mutants (Keio collection) as donors and a *Saccharomyces cerevisiae* strain as a recipient. Out of 3884 mutants, two mutants (∆*aceE*, ∆*priA*) showed a severe decrease in TKC efficiency by more than two orders of magnitude but not in bacterial conjugation. The effect on TKC efficiency by the two mutants was partly recovered by a preculture with a fresh culture medium before the TKC reaction, regardless of the presence of antibiotics. These results suggest that no single chromosomal target gene is solely responsible for universally blocking IncP1-type conjugation by impeding its function. The results also suggest the existence of an unidentified recognition or transfer mechanism distinct from bacterial conjugation, highlighting the novel roles of *aceE* and *priA*.

## 1. Introduction

Conjugal transfer is one of the major driving forces of horizontal gene transfer in eubacteria, as well as transformation and transduction [1]. The genes necessary for conjugal transfer are generally encoded either on plasmids or integrative and conjugative elements, and the genes are mainly classified into two groups. The first group consists of genes for DNA transfer and replication (Dtr) proteins, which form the relaxosome at the origin of transfer (*oriT*), and the second group consists of genes for type IV secretion system (T4SS) proteins, which form the mating channel between donor and recipient cells [2]. In the case of the IncP1-type plasmid, the genes for the Dtr proteins are encoded on the *tra* gene cluster, and those for T4SS proteins are encoded on *trb* [3].

The host range of a self-transmissible plasmid is restricted based on its transfer ability and replication ability in a recipient bacterium. This means that the T4SS derived from a broad-host-range plasmid has the ability to transfer across a broad range of hosts. The IncP1-type plasmid has the ability to be transferred and replicated in hosts belonging to at least three proteobacteria subclasses: Alphaproteobacteria [4,5], Betaproteobacteria [6,7], and Gammaproteobacteria [4,7,8,9]. In 1989, Heinemann and Sprague found that conjugative transfer systems from an IncP1 and an IncF plasmid were capable of transferring DNA even from *E. coli* to a eukaryote, *Saccharomyces cerevisiae* [10]. Although the transfer by the IncF plasmid has not been reproduced for long, that by the IncP1 plasmid has been studied by several research groups, and it came to be called trans-kingdom conjugation (TKC) [11,12,13,14]. In addition to yeasts, its broad transfer ability has been reported in mammalian cell cultures [15], archaebacteria (inter-domain conjugation) [16,17], and diatoms [18,19,20], although some of their reproducibility and applicability need to be confirmed by independent research groups before being accepted as a general gene introduction tool, except in *S. cerevisiae*.

Recently, based on the potential applicability of TKC as a gene introduction tool for eukaryotic and archaeal cells, improvements in TKC vector and helper plasmid constructs and methods have been tried, especially in *S. cerevisiae*. Vectors for yeast two-hybrid screening, which are available for prey library construction using TKC, and a TKC vector that enables the removal of unnecessary accessory DNA for recipients during transfer, have been reported [21]. TKC vectors, which were stably maintained not only in *E. coli* but also in *Sinorhizobium meliloti*, were constructed and successfully transferred to *S. cerevisiae* and *Phaeodactylum tricornutum* [22]. More recently, high TKC-efficiency vectors, which are also applicable as helper plasmids, were developed by mutating the promoter region of *traJ* derived from an IncP1α plasmid, RP4/RK2. Interestingly, the enhancement of DNA transfer was observed in all three examined fungal species, but not in *E. coli* [23]. TKC DNA transfer occurred at a practical use frequency by simply mixing overnight cultures of donor *E. coli* and recipient *S. cerevisiae* derived from industrial and wild strains [24]. A TKC method, in which the reaction was performed within solid media, achieved stable DNA transfer of longer than 100 kb DNA into *S. cerevisiae* [25].

In contrast to the recent advancements in TKC tools and methods for practical use, the analysis of the TKC mechanism has lagged behind. To address this, we previously performed genome-wide screenings using a set of knockout mutants that covered all nonessential genes. Our findings suggested that genes primarily responsible to vacuolar-type ATPase were expected to positively affect TKC, while those responsible to mitochondrial F1F0 ATPase were expected to negatively affect TKC [26,27]. However, the detailed mechanism remains unclear because these two ATPases have not previously been recognized as factors influencing DNA incorporation and/or cell permeability. Recently, we also reported on donor genes that negatively affected both bacterial and trans-kingdom conjugations using a set of knockout mutants that covered every nonessential gene in *E. coli* (Keio collection). The genes (*frmR*, *sufA,* and *iscA*) genetically interacted with each other, and knockout mutants of their orthologous genes in *Agrobacterium tumefaciens* also showed an increase in TKC efficiency, suggesting that the negative effect of these genes was conserved among eubacteria [28]. However, donor genes that positively affect trans-kingdom conjugation have not yet been identified. Once the remaining donor genes that positively affect TKC are identified, we will have a dataset of genes that positively and negatively affect TKC in both donor and recipient organisms. Subsequent analysis using combinations of these mutant strains will clarify the underlying mechanisms of TKC. These insights may enable the targeted engineering/selection of donor bacteria to optimize TKC as a gene introduction tool for eukaryotes.

In this study, we identified genes in the *E. coli* genome that positively affected TKC through a T4SS encoded by the RP4 plasmid (RP4-T4SS) using an *E. coli* single-knockout mutant donor library.

## 2. Materials and Methods

### 2.1. Bacterial Strains, Yeast, and Growth Media

The *E. coli* and *S. cerevisiae* strains used in this study are listed in Table 1. The knockout mutant collection, which contains pRS316∷*oriT*^P^ (a TKC vector) and pRH220 (a helper plasmid), was derived from a complete set of *E. coli* nonessential gene deletion clones (Keio collection) and was prepared in our previous work [28,29]. *E. coli* strains were routinely cultured in LB Lennox medium (Becton Dickinson, Franklin Lakes, NJ, USA) at 37 °C, and at 28 °C if necessary. In addition, *S. cerevisiae* was cultured in a yeast-extract/peptone/dextrose (YPD) medium (Becton Dickinson, Franklin Lakes, NJ, USA). A synthetic defined medium (Becton Dickinson, Franklin Lakes, NJ, USA) containing appropriate individual amino acids (leucine, 0.03 mg/mL; histidine, 0.02 mg/mL; and lysine, 0.03 mg/mL) was used as the selection media (SC−Ura) for yeast transconjugants at 28 °C. A solid LB Lennox medium was prepared by the addition of 1.5% agar, and solid YPD and SC−Ura media were prepared by the addition of 2% agar. Antibiotics, including chloramphenicol (Chl; 30 μg/mL), ampicillin (Amp; 50 μg/mL), kanamycin (Kan; 50 μg/mL), and tetracycline (Tet; 7.5 μg/mL), were supplemented as necessary. All amino acids and other chemicals were purchased from Fujifilm Wako Pure Chemical (Osaka, Japan).

### 2.2. Donor and Recipient Cell Cultures

The plasmids used in this study are listed in Table 2. For the genome-wide screening analysis, donor *E. coli* Keio mutant strains carrying pRH220 and pRS316∷*oriT*^P^ were inoculated from 96-well frozen stock plates using a pin replicator to 96-well flat-bottom plates and cultured in 100 µL of medium supplemented with Amp, Chl, and Kan at 37 °C for 15 to 18 h. The cultures were directly used for the TKC reaction in the first- and second-round screenings. From the third- to fifth-round screenings, 50 µL of the overnight cultures was inoculated into 100 µL of fresh medium and cultured for 1 h at 37 °C. At the sub-screening step after the fifth-round screening, the same method as for the first- and second-round screenings was applied, but Kan was excluded, and the culture scale was increased to 1 mL. In the final-round screening and subsequent experiments, the candidate strains were first prepared on solid medium plates, then inoculated into 5 mL of liquid medium in a tube and cultured for 15 to 18 h.

The recipient strain of *S. cerevisiae* BY4742 was cultured in 5 mL of liquid medium in a tube for 18 to 22 h at 28 °C. Both donor and recipient liquid cultures were cultured with agitation to allow aeration.

### 2.3. Trans-Kingdom Conjugation

**Figure 1:**
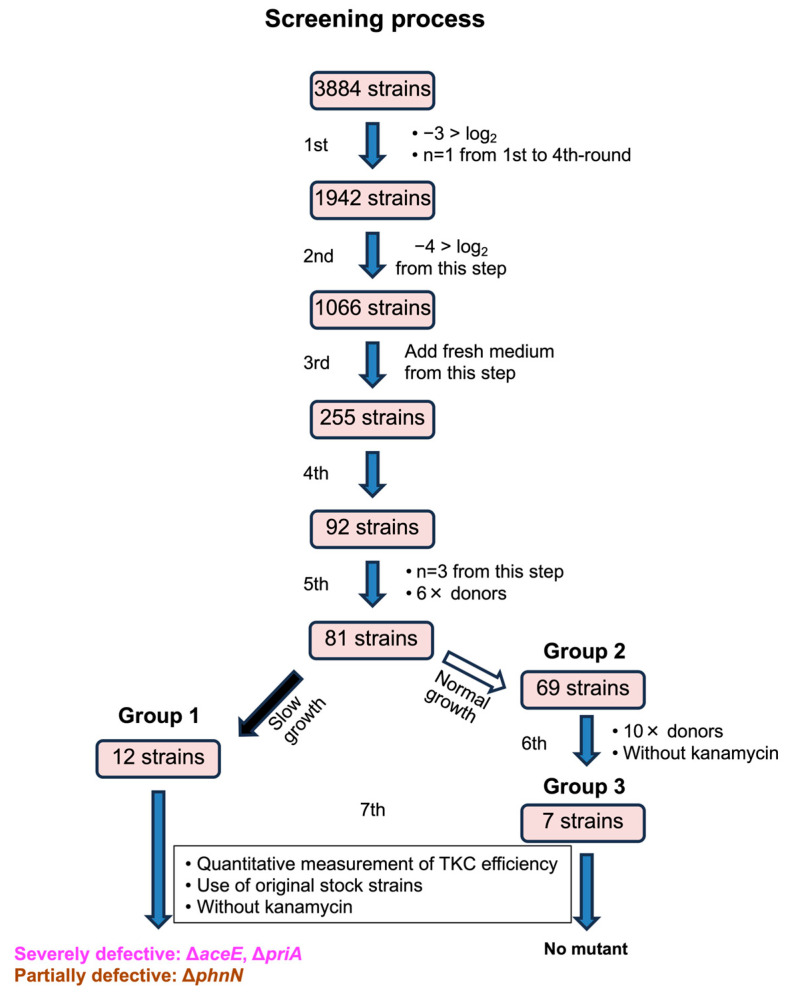
Overall process of the genome-wide screening. The screening process is illustrated in a flow chart. From 3884 knockout mutants, two mutants (∆*aceE*, ∆*priA*) were isolated. The details are described in the Supplementary Materials.

The screening process is summarized in Figure 1. In the first- and second-round screenings, 50 µL of each donor overnight culture and 50 µL (approximately 1 × 10^6^ cfu) of yeast recipient suspended in TNB (80 mM Tris-HCl [pH 7.5], and 0.05% NaCl) were mixed and incubated at 28 °C for 1 h, followed by the selection of transconjugants by spotting 15 µL of the conjugation reaction onto SC−Ura supplemented with Tet. The culture plate was incubated for 48 to 72 h at 28 °C. In the third- to fifth-round screenings, 150 µL of each donor-inoculated culture was concentrated by 6-fold, and 25 µL of the culture and 25 µL of the recipient suspension were mixed to perform the TKC reaction. The spotting volume for these screening stages was 10 µL. After the fifth-round screening, the remaining candidate strains were separated into two groups: group 1 (12 strains, mainly including slow-growing strains) and group 2 (69 strains). In the case of group 2, further screening was performed by using 25 µL of each 10-fold concentrated donor suspension in TNB without antibiotics, mixed with 25 µL of the recipient suspension (sub-screening). For the remaining strains after the sub-screening and the group 1 strains, the TKC reaction was performed with adjusted donor turbidity (OD_660_ = 1.8). Then, 250 µL of the donor suspension in TNB without antibiotics and 250 µL of the recipient suspension were mixed to perform the TKC reaction (standard scale TKC reaction in this report). At this stage, in addition to plating the reaction mixture on a solid SC−Ura plate supplemented with Tet, plating on a YPD plate supplemented with Tet and/or on an LB Lennox plate supplemented with Kan and Amp was performed to calculate TKC efficiency.

The turbidity of the donor and recipient cultures was measured using a microtiter-plate reader MTP-310 (Corona, Ibaraki, Japan).

### 2.4. Bacterial Conjugation

We used a protocol identical to the one used for the TKC (standard scale TKC reaction), where 250 µL of SY327 (λ*pir*) recipient suspensions in TNB (OD_660_ = 1.8) was used. The conjugation reaction was performed for 1 h. The transconjugants were selected on LB Lennox solid medium supplemented with rifampicin and the appropriate antibiotics for the selection of the transferred plasmid.

### 2.5. Complementation Analysis

Two *E. coli* KO mutants, Δ*aceE* and Δ*priA*, derived from the Keio collection, were transformed with pJP5603sacBGmR (+*aceE* or *priA*, including each adjacent sequence) via conjugation by S17-1 λ*pir*. Primary homologous recombination into the genomes of the Δ*aceE* and Δ*priA* strains was selected based on gentamicin resistance (30 μg/mL). The selection of the secondary homologous recombination was performed by culturing the strains on LB Lennox medium containing 10% sucrose. The successfully complemented strains, with the complete removal of the Kan resistance gene cassette, were selected by Kan sensitivity on LB Lennox media plates with and without Kan, followed by the introduction of pRH220 and pRS316∷*oriT*^P^. The assessment of the conjugation efficiency (within 1 h of co-cultivation) by these complemented strains, in comparison to wild-type and single-KO mutants of Δ*aceE* and Δ*priA*, was performed.

### 2.6. Data Analysis

During the screening step, relative TKC efficiency was measured following our previous report [30] with some modifications. In brief, the number of transconjugant colonies for each mutant was divided by the relative turbidity value of the corresponding input donor culture and defined as the transconjugant colony value (TCV). Then, using the control strain BW25113 carrying pBBR122Δ*Cm*^R^ in addition to the TKC vector and helper plasmids, the median TCV (MTCV) of seven control reactions in each conjugation experiment set (defined as the MTCV_ctrl_) was calculated. The log_2_ value of the relative TCV (RTCV = TCV/MTCV_ctrl_) was defined as an arbitrary unit and calculated. At the fifth-round screening, the experiment was repeated three times, and the average of the RTCV was applied.

At and after the final screening stage, the absolute value of the conjugation efficiency (transconjugants/output recipient and/or transconjugant/output donor) was calculated for each mutant. Data were expressed as the mean ± standard deviation (SD) of at least three independent biological experiments. Statistical analyses were performed using either Microsoft Excel for Microsoft 365 MSO (version 2501, Microsoft) or the public-domain R program (version 4.3.1). Results were considered statistically significant when *p* < 0.05.

## 3. Results

### 3.1. Screening of Chromosomal Mutants Defective in TKC

**Figure 2:**
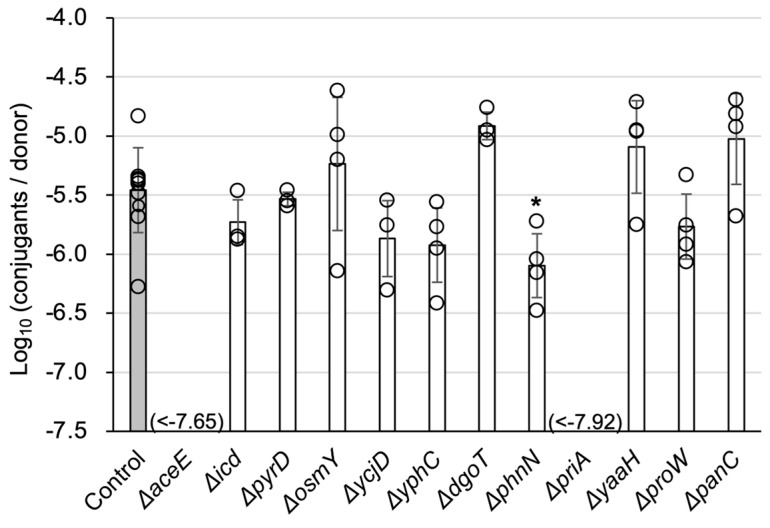
∆*aceE* and ∆*priA* mutants are severely defective in TKC. The result of the final screening step in group 1 is shown as an example of the screening results. Data are expressed as the mean ± standard deviation (SD) of at least three independent experimental replicates. An asterisk (*) indicates a statistically significant difference against the control at *p* < 0.05 (two-tailed *t*-test).

The whole screening process is summarized in Figure 1. The relative TKC efficiency of mutant strains tended to be lower than that of the parental strain and was widely distributed. Thus, we determined that the detection accuracy of the screening was insufficient and conducted multiple rounds of screening to eliminate false-positive (false TKC-deficient) mutant strains. Additional details are provided in the Supplementary Materials. Finally, two mutants (∆*aceE*, ∆*priA*) were isolated as mutants with severely decreased TKC efficiency, at an undetectable level, at least by more than two orders of magnitude (Figure 2). The *aceE* gene encodes a subunit of the E1p component of the pyruvate dehydrogenase complex, while *priA* encodes an N′ protein of the primosome (EcoCyc database; https://ecocyc.org/ last accessed on 17 February 2025).

### 3.2. Confirmation of TKC Defectiveness in ∆aceE and ∆priA Mutants

**Figure 3:**
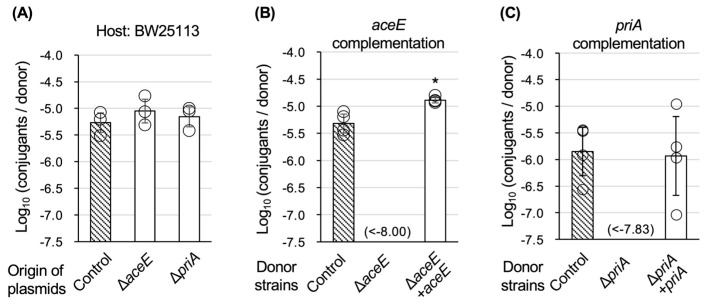
Confirmation analyses that *aceE* and *priA* genes are the chromosomal genes responsible for TKC. (**A**) Normality check of TKC ability in strains that had reintroduced the helper and TKC vector plasmids collected from the two defective mutants into the parental strain BW25113. (**B**,**C**) Recovery check of TKC ability in each defective strain by complementing the respective knockout genes (*aceE* in **B** and *priA* in **C**). The combinations “∆*aceE + aceE*” and “∆*priA + priA*” represent complemented strains. Three, four, and four independent experimental replicates were performed in **A**, **B**, and **C**, respectively. Data are presented as the mean ± SD. An asterisk (*) indicates a statistically significant difference against the control at *p* < 0.05 (two-tailed *t*-test).

During our screening process, a change in some mutant strains to a TKC-deficient phenotype was observed. Therefore, additional experiments were performed to carefully confirm that the two genes were the genes responsible for TKC on the chromosome. First, the helper plasmid and TKC vector were extracted from the ∆*aceE* and ∆*priA* strains and reintroduced into the parental strain, BW25113. The reintroduced strains showed normal TKC ability, proving that the helper plasmid and TKC vector were normal (Figure 3A). Next, the two KO mutant strains were complemented by the respective deleted genes, and the TKC ability was examined. The TKC ability was restored in each complemented strain (Figure 3B,C). These results demonstrated that the two genes, *aceE* and *priA*, are the genes responsible for positive factors of TKC on the donor *E. coli* chromosome.

### 3.3. Characterization of TKC Defectiveness in ∆aceE and ∆priA Mutants

**Figure 4:**
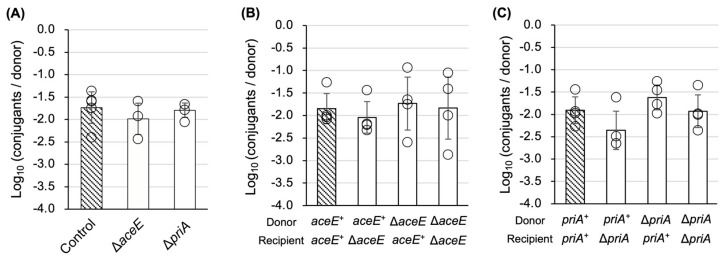
Analyses of conjugation ability of the two mutants in bacterial conjugation. (**A**) Conjugal transfer analysis from ∆*aceE* and ∆*priA* mutants to *E. coli* SY327 (λ*pir*). (**B**,**C**) Bacterial conjugation ability check between ∆*aceE* mutants (**B**) and between ∆*priA* mutants (**C**). Donors are BW25113 (shown as *aceE*^+^ in (**B**) and *priA^+^* in (**C**)) and its ∆*aceE* and ∆*priA* mutants, while recipients are SY327 (λ*pir*) (shown as *aceE^+^* in (**B**) and *priA^+^* in (**C**)) and its ∆*aceE* and ∆*priA* mutants. Three or four, four, and four independent experimental replicates were performed in **A**, **B**, and **C**, respectively. Data are presented as the mean ± SD.

To assess the generality of the deficiency caused by these two mutations on conjugal transfer, a bacterial conjugation analysis between *E. coli* strains was performed. Unexpectedly, both ∆*aceE* and ∆*priA* showed no deficiency in bacterial conjugation (Figure 4A). Based on this result, we hypothesized that materials lacking in the donor mutant cells were supplied by the recipient cells and complemented the donor deficiency in conjugation. To confirm this possibility, we performed bacterial conjugation between ∆*aceE* mutants and between ∆*priA* mutants, respectively. However, both experiments did not show a deficiency in conjugation (Figure 4B,C). Therefore, the conjugation deficiency caused by the absence of the two genes is a TKC-specific phenomenon.

There have been no reports of chromosomal factors that act positively on TKC. In addition, the gene products of *aceE* and *priA* are unrelated to each other and to the factors that act negatively on TKC, which we identified previously [28]. Thus, we could not speculate on the mechanism of action of these gene products on TKC. To obtain clues, we therefore examined the TKC ability of genes related to *aceE* and *priA*.

**Figure 5:**
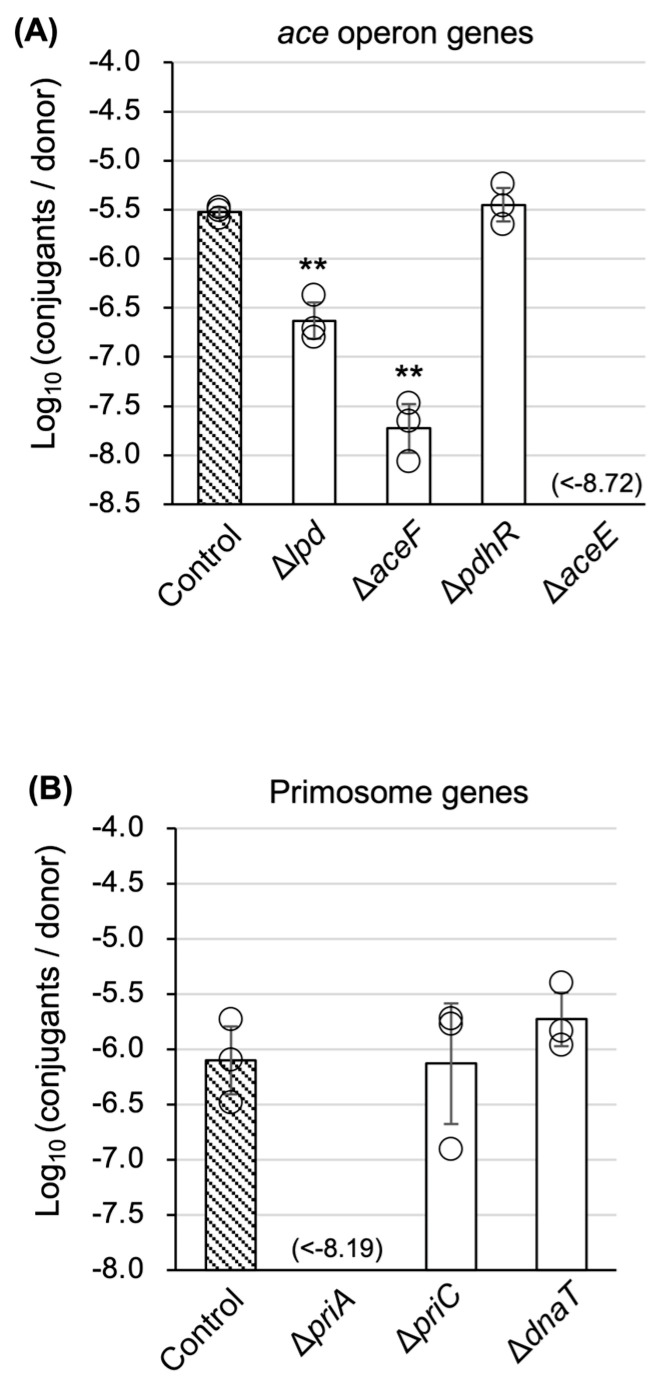
Analyses of TKC ability in mutants included in the *ace* operon (**A**) and primosome genes (**B**). Each analysis was performed in triplicate. Data are presented as the mean ± SD. Asterisks (**) indicate a statistically significant difference against the control at *p* < 0.01 (two-tailed *t*-test).

The pyruvate dehydrogenase complex, which metabolizes pyruvate to acetyl-CoA, consists of three subunits, E1, E2, and E3, and the corresponding *E. coli* genes are *aceE*, *aceF*, and *lpd*. These genes form an operon and are negatively regulated by a repressor, PdhR. As shown in Figure 5A, a decrease in TKC efficiency by one to two orders of magnitude was observed in the **∆***aceF* and **∆***lpd* mutants, but not a severe decrease as seen in the ∆*aceE* mutant. In addition, mutants of other genes for the pyruvate metabolism to acetyl-CoA showed no significant decrease in TKC (Figure S5). These results indicate that the inhibition of the pyruvate-to-acetyl-CoA metabolic pathway itself does not cause defects in TKC ability and that AceE (E1 subunit) has an unknown function.

The primosome is mainly involved in the restart of stalled replication forks and consists of six subunits. Among the corresponding genes for these subunits, *priA* and two other genes, *priC* and *dnaT*, are known to be nonessential for survival, so we examined the TKC ability using knockout mutants of the two genes. No significant decrease was observed (Figure 5B). PriA (N′ subunit) also seems to have an unknown function in TKC, independent of its role as a subunit of the primosome.

### 3.4. Culture Medium Substitution Recovers TKC Defectiveness in ∆aceE and ∆priA Mutants

**Figure 6:**
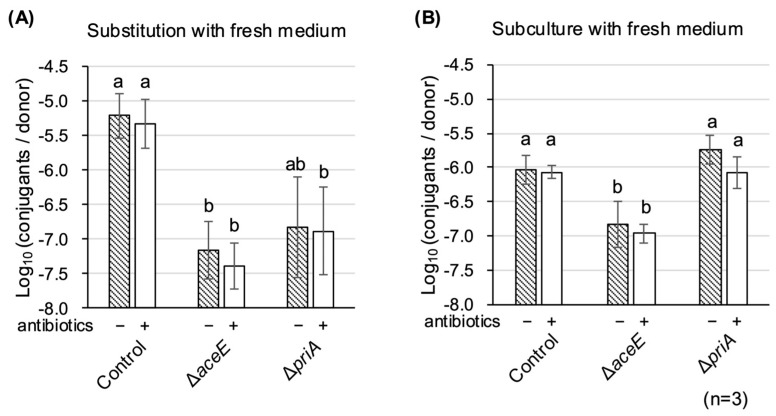
Effect of preculture with fresh medium before TKC reaction on ∆*aceE* and ∆*priA* mutants. TKC analysis of the two mutants with a preculture of donor cells by substituting the same amount of fresh medium (**A**) or by resuspending and diluting the collected donor cells at OD_600_ = 0.09 (**B**). Shaded and white bars indicate the presence or absence of antibiotics (chloramphenicol and ampicillin), respectively, in the subculture medium. Each analysis was performed in triplicate. Data are presented as the mean ± SD. Different letters indicate significant differences at *p* < 0.05 using Tukey HSD multiple comparison analysis.

Zhang et al. reported the influence of antibiotics on RP4-T4SS bacterial conjugation efficiency [30]. Therefore, although bacterial conjugation was normal, we examined the effect of antibiotics on TKC using the two mutant strains. The donor strains were precultured with fresh medium with or without Amp and Chl before the TKC reaction. Unexpectedly, a detectable recovery of TKC was observed in both strains, regardless of the presence of antibiotics, when the cells were collected from overnight culture medium and simply substituted with the same amount of fresh LB medium (Figure 6A). When the cells were collected and diluted with fresh medium, the ∆*priA* mutant recovered to a comparable level to the parental control strain (Figure 6B).

## 4. Discussion

In this study, we successfully isolated two chromosomal gene mutants that showed decreased TKC efficiency through a genome-wide screening. In our previous study, three genetically linked chromosomal gene mutants showing increased TKC efficiency were smoothly enriched after three rounds of screening [28]. In contrast, the screening for deficient mutants was difficult due to the inclusion of an unexpected number of false-positive (false TKC-deficient) mutants (see Supplementary Materials). Therefore, only the mutants that showed severe TKC deficiency were isolated, but we were unable to establish a screening method that was both accurate and high-throughput. The donor library should still contain mutants with a less severe decrease in TKC efficiency, such as the ∆*aceF* and ∆*lpd* mutants identified in the subsequent experiment. Interestingly, the screened ∆*aceE* and ∆*priA* mutants showed a lack of conjugation ability in TKC but not in bacterial conjugation. This result may indicate that conjugative transfer between *E. coli* strains via the RP4-T4SS is sufficiently adaptive to accommodate a wide range of mutations in the donor strain, as we reported in a similar study with recipient mutants [29]. However, we should not exclude the possible existence of mutants that are specifically deficient in bacterial conjugation.

The *aceE* gene encodes the E1 subunit of the pyruvate dehydrogenase (PDH) multienzyme complex. This complex also includes the E2 and E3 subunits, which are encoded by *aceF* and *lpd*, respectively. These genes are included in one operon, which is regulated by a transcriptional regulator, PdhR [31]. The E1 subunit catalyzes the first step of the reaction: the decarboxylation of pyruvate and the reductive acetylation of the lipoyl group bound to the E2 subunit [32]. In *aceF* and *lpd* mutants, TKC was not completely blocked (Figure 5A). In addition, no decrease in TKC efficiency was observed in mutants of genes involved in other metabolic pathways from pyruvate to acetyl-CoA (Figure S5). These results suggest that the inhibition of the pyruvate-to-acetyl-CoA metabolism itself does not inhibit TKC, and that the AceE protein is involved in TKC through an unknown function other than the pyruvate metabolism. The decreased efficiency of TKC in ∆*aceF* and ∆*lpd* mutants is presumably due to the inhibition of the AceE function caused by the deficiency in these genes, rather than the direct involvement of these gene products in TKC. Another interpretation of the lack of TKC ability in the ∆*aceE* mutant is that the absence of AceE causes the highest level of pyruvate accumulation among the pyruvate dehydrogenase-related mutants, which influences the expression of genes regulated by PdhR. PdhR activity is controlled by pyruvate, and a recent study showed that PdhR was a bifunctional global regulator controlling a total of 16–23 targets, including not only the genes involved in central carbon metabolism but also some genes for the surrounding pyruvate-sensing cellular pathways such as fatty acid degradation and flagella formation [33]. However, this interpretation can be eliminated because the ∆*pdhR* mutant did not show a significant change in TKC, in which the expression status of PdhR-regulated genes should be similar to that in ∆*aceE* (Figure 5A).

PriA is a component of the primosome, consisting of six types of proteins: PriA, PriB, PriC, DnaB, DnaG, and DnaT. The primosome is generally known to function in the restart of stalled replication forks. Although it is also known to function in the initiation of replication in various plasmids and phages [34], the relationship between the primosome, especially PriA, and conjugation has been poorly reported and understood. Alalam et al. recently reported that the ∆*priA* donor strain derived from the Keio mutant showed no measurable conjugation in the F plasmid [35]. In our previous study, we examined bacterial conjugation ability in the ∆*priA* Keio mutant strain using the identical helper (pRH220) and TKC vector (pRS316∷*oriT*^P^) combination used in this study. ColE1-type plasmid replication is known to be inhibited in *priA* mutants [36], and the TKC vector included a ColE1-type origin of replication. Unexpectedly, the ∆*priA* recipient did not show conjugation deficiency, although the growth of the transconjugant was very slow [29]. This result indicates that the acceptance of transferred plasmid DNA is not inhibited in the ∆*priA* recipient. In this study, the conjugation ability of the ∆*priA* donor was decreased in TKC, but not in bacterial conjugation (Figure 2 and Figure 4A,C).

An interpretation of this result is that PriA or uncharacterized factor(s) might be necessary for plasmid transfer, and the factor might be exchanged between donor and recipient cells. In this case, when bacterial conjugation occurs between a ∆*priA* donor and a *priA*^+^ recipient, the factor can be supplied from the recipient cell, and the conjugation proceeds normally. However, when TKC occurs between a ∆*priA* donor and a yeast recipient, the yeast cell cannot complement the lack of the factor. However, the bacterial conjugation efficiency between the ∆*priA* donor and recipient, as well as between the ∆*aceE* donor and recipient, was normal (Figure 4B,C). Therefore, we can eliminate the interpretation that the supplement of required factor(s) in the two deficient donors from a normal recipient occurred. Another interpretation is that some cell–cell interaction between *E. coli* and *S. cerevisiae*, independent of the RP4-T4SS pilus, might be important for TKC, and the ∆*priA* donor may have a deficiency in the interaction caused by a change in cell surface composition.

**Figure 7:**
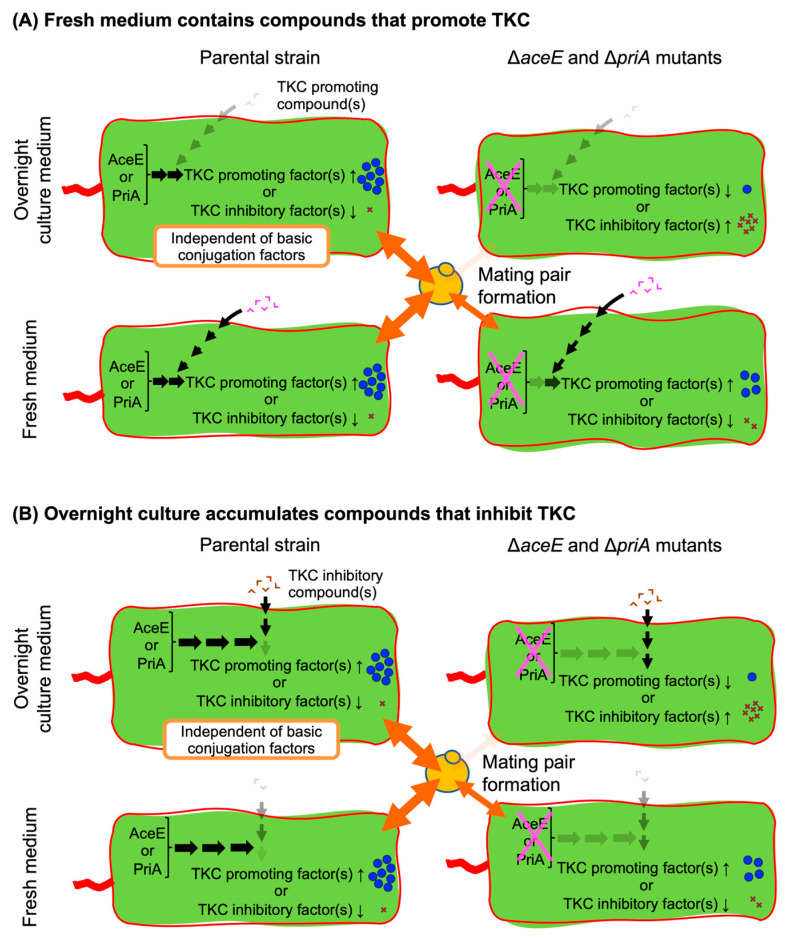
Proposed TKC models involving the novel functions of *aceE* and *priA* genes. (**A**) Model illustrating the scenario where fresh medium contains compounds that promote TKC. (**B**) Model illustrating the scenario where overnight culture accumulates compounds that inhibit TKC. These mechanisms are independent of those involved in basic conjugation, because bacterial conjugation ability remains normal in ∆*aceE* and ∆*priA* mutants.

In summary, based on the results so far, we propose TKC models involving a novel function of the *aceE* and *priA* genes (Figure 7). If we assume that fresh medium contains compounds that promote TKC, AceE and PriA are thought to function as inducing factors that promote TKC or suppressing factors that inhibit TKC. This action ensures that the donor *E. coli* and the recipient yeast have the necessary interactions for TKC. AceE and PriA can promote TKC regardless of the presence or absence of TKC-promoting compounds. Conversely, TKC-promoting compounds can promote TKC downstream of AceE and PriA, so even ∆*aceE* and ∆*priA* mutants retain their TKC ability under fresh medium culture conditions (Figure 7A). Alternatively, if we assume that TKC-inhibiting compounds accumulate in the overnight culture, AceE and PriA are thought to have the role of blocking TKC inhibition by TKC-inhibiting compounds. Therefore, ∆*aceE* and ∆*priA* mutants are suppressed in TKC under overnight culture conditions (Figure 7B). We would like to add that other possible models exist, and further verification is required.

## Figures and Tables

**Table 1:** Strains used in this study.

Strains	Relevant Characteristics	Source or Reference
*E. coli*		
Keio collection	An in-frame single-gene knockout mutant collection derived from BW25113, Kan^R^	NBRP Japan
BW25113	*F^−^ Δ(araD-araB)567 ΔlacZ4787(*∷*rrnB-3)* *λ-rph-1 Δ(rhaD-rhaB)568 hsdR514*	NBRP Japan
SY327 (*λpir)*	*Δ(lac pro) argE*(Am) *recA56 Rif*^R^ *Nal*^R^ λ*pir*, Rif^R^ Nal^R^	NBRP Japan
SY327 (*λpir) ΔaceE*	*Δ(lac pro) argE*(Am) *recA56 Rif*^R^ *Nal*^R^ λ*pir ΔaceE*, Rif^R^ Nal^R^ Kan^R^	This study
SY327 (*λpir) ΔpriA*	*Δ(lac pro) argE*(Am) *recA56 Rif*^R^ *Nal*^R^ λ*pir ΔpriA*, Rif^R^ Nal^R^ Kan^R^	This study
S17-1 (*λpir)*	*F^−^ RP4-2(Kan*^R^∷*Tn7 Tet*^R^∷*Mu-1) pro-82 λpir recA1 endA1 thiE1 hsdR17 creC510*	NBRP Japan
*S. cerevisiae*		
BY4742	*MAT*α *SSD1-V his3∆1 leu2∆0 lys2∆0 ura3∆0*	Invitrogen, Carlsbad, CA

**Table 2:** Plasmids used in this study.

Plasmids	Relevant Characteristics	Source or Reference
pJP5603sacBGmR	Mobilizable plasmid; *sacB oriT* ^RP4^ *Gen*^R^ Used for the construction of *E. coli* complementation strains	Zoolkefli et al., 2021 [28]
pBBR122∆*Cm*^R^	Derivative of a commercially provided plasmid vector pBBR122; *mob*^pBBR1^′ (non-transmissible) *Kan*^R^ *∆Chl*^R^	Moriguchi et al., 2020 [29]
RP4	IncP1α-type conjugative broad-host-range plasmid; *Kan*^R^ *Tet*^R^ *Amp*^R^	Pansegrau et al., 1994 [3]
pRH220	Helper plasmid; *tra*^RP4^ *trb*^RP4^ *oriT*^RP4^ *ori*-pSC101 *Chl*^R^	* AB526840
pRS316∷*oriT*^P^	Mobilizable plasmid; *URA3 CEN6/ARSH4 ori*-pMB1 *Amp*^R^ *oriT*^RP4^	Moriguchi et al., 2013 [21]

* DDBJ/EMBL/GenBank accession number.

## Data Availability

All figures and tables are contained within the article and Supplementary Materials. The numerical data on which they are based are available upon request to the corresponding author.

## Supplementary Materials

The following supporting information can be downloaded at: https://www.mdpi.com/article/10.3390/microorganisms13030488/s1, Supplementary Materials_microorganisms: Detailed description of the genome-wide screening process and TKC ability in mutants of other genes involved in pyruvate metabolism to acetyl-CoA. This file includes Figures S1–S5.

## Abbreviations

The following abbreviations are used in this manuscript:

TKC	Trans-kingdom conjugation
NBRP	National BioResource Project
SD	Standard deviation
TNB	Tris-HCl with NaCl buffer
YPD	Yeast-extract/peptone/dextrose

## References

[1] Furuya E.Y., Lowy F.D. (2006). Antimicrobial-resistant bacteria in the community setting. Nat. Rev. Microbiol..

[2] Costa T.R.D., Harb L., Khara P., Zeng L., Hu B., Christie P.J. (2021). Type IV secretion systems: Advances in structure, function, and activation. Mol. Microbiol..

[3] Pansegrau W., Lanka E., Barth P.T., Figurski D.H., Guiney D.G., Haas D., Helinski D.R., Schwab H., Stanisich V.A., Thomas C.M. (1994). Complete nucleotide sequence of birmingham IncPα plasmids: Compilation and comparative analysis. J. Mol. Biol..

[4] Schmidhauser T.J., Helinski D.R. (1985). Regions of broad-host-range plasmid RK2 involved in replication and stable maintenance in nine species of Gram-negative bacteria. J. Bacteriol..

[5] Yano H., Rogers L.M., Knox M.G., Heuer H., Smalla K., Brown C.J., Top E.M. (2013). Host range diversification within the IncP-1 plasmid group. Microbiology.

[6] Kamachi K., Sota M., Tamai Y., Nagata N., Konda T., Inoue T., Top E.M., Arakawa Y. (2006). Plasmid pBP136 from *Bordetella pertussis* represents an ancestral form of IncP-1β plasmids without accessory mobile elements. Microbiology.

[7] Suzuki H., Yano H., Brown C.J., Top E.M. (2010). Predicting plasmid promiscuity based on genomic signature. J. Bacteriol..

[8] Norberg P., Bergström M., Jethava V., Dubhashi D., Hermansson M. (2011). The IncP-1 plasmid backbone adapts to different host bacterial species and evolves through homologous recombination. Nat. Commun..

[9] Adamczyk M., Jagura-Burdzy G. (2003). Spread and survival of promiscuous IncP-1 plasmids. Acta Biochem. Pol..

[10] Heinemann J.A., Sprague G.F. (1989). Bacterial conjugative plasmids mobilize DNA transfer between bacteria and yeast. Nature.

[11] Nishikawa M., Suzuki K., Yoshida K. (1990). Structural and functional stability of IncP plasmids during stepwise transmission by trans-kingdom mating: Promiscuous conjugation of *Escherichia coli* and *Saccharomyces cerevisiae*. Jpn. J. Genet..

[12] Hayman G.T., Bolen P.L. (1993). Movement of shuttle plasmids from *Escherichia coli* into yeasts other than *Saccharomyces cerevisiae* using trans-kingdom conjugation. Plasmid.

[13] Sawasaki Y., Inomata K., Yoshida K. (1996). Trans-kingdom conjugation between *Agrobacterium tumfaciens* and *Saccharomyces cerevisiae,* a bacterium and a yeast. Plant Cell Physiol..

[14] Bates S., Cashmore A.M., Wilkins B.M. (1998). IncP plasmids are unusually effective in mediating conjugation of *Escherichia coli* and *Saccharomyces cerevisiae*: Involvement of the Tra2 mating system. J. Bacteriol..

[15] Waters V.L. (2001). Conjugation between bacterial and mammalian cells. Nat. Genet..

[16] Dodsworth J.A., Li L., Wei S., Hedlund B.P., Leigh J.A., de Figueiredo P. (2010). Interdomain conjugal transfer of DNA from bacteria to archaea. Appl. Environ. Microbiol..

[17] Fink C., Beblawy S., Enkerlin A.M., Mühling L., Angenent L.T., Molitor B. (2021). A shuttle-vector system allows heterologous gene expression in the thermophilic methanogen *Methanothermobacter thermautotrophicus* ΔH. mBio.

[18] Karas B.J., Diner R.E., Lefebvre S.C., McQuaid J., Phillips A.P.R., Noddings C.M., Brunson J.K., Valas R.E., Deerinck T.J., Jablanovic J. (2015). Designer diatom episomes delivered by bacterial conjugation. Nat. Commun..

[19] Awwad F., Fantino E.I., Héneault M., Diaz-Garza A.M., Merindol N., Custeau A., Gélinas S.E., Meddeb-Mouelhi F., Li J., Lemay J.F. (2023). Bioengineering of the marine diatom *Phaeodactylum tricornutum* with cannabis genes enables the production of the cannabinoid precursor, olivetolic acid. Int. J. Mol. Sci..

[20] Maeda Y., Nakamura M., Watanabe K., Okamoto E., Tanaka T. (2023). Functional analysis of the putative centromere sequences of marine oleaginous diatom *Fistulifera solaris*. Algal Res..

[21] Moriguchi K., Edahiro N., Yamamoto S., Tanaka K., Kurata N., Suzuki K. (2013). Transkingdom genetic transfer from *Escherichia coli* to *Saccharomyces cerevisiae* as a simple gene introduction tool. Appl. Environ. Microbiol..

[22] Brumwell S.L., MacLeod M.R., Huang T., Cochrane R.R., Meaney R.S., Zamani M., Matysiakiewicz O., Dan K.N., Janakirama P., Edgell D.R. (2019). Designer *Sinorhizobium meliloti* strains and multi-functional vectors enable direct inter-kingdom DNA transfer. PLoS ONE.

[23] Cochrane R.R., Shrestha A., Severo De Almeida M.M., Agyare-Tabbi M., Brumwell S.L., Hamadache S., Meaney J.S., Nucifora D.P., Heng Say H., Sharma J. (2022). Superior conjugative plasmids delivered by bacteria to diverse fungi. BioDesign Res..

[24] Moriguchi K., Yamamoto S., Ohmine Y., Suzuki K. (2016). A fast and practical yeast transformation method mediated by *Escherichia coli* based on a trans-kingdom conjugal transfer system: Just mix two cultures and wait one hour. PLoS ONE.

[25] Soltysiak M.P.M., Meaney R.S., Hamadache S., Janakirama P., Edgell D.R., Karas B.J. (2019). Trans-kingdom conjugation within solid media from *Escherichia coli* to *Saccharomyces cerevisiae*. Int. J. Mol. Sci..

[26] Mizuta M., Satoh E., Katoh C., Tanaka K., Moriguchi K., Suzuki K. (2012). Screening for yeast mutants defective in recipient ability for transkingdom conjugation with *Escherichia coli* revealed importance of vacuolar ATPase activity in the horizontal DNA transfer phenomenon. Microbiol. Res..

[27] Moriguchi K., Yamamoto S., Tanaka K., Kurata N., Suzuki K. (2013). Trans-kingdom horizontal DNA transfer from bacteria to yeast is highly plastic due to natural polymorphisms in auxiliary nonessential recipient genes. PLoS ONE.

[28] Zoolkefli F.I.R.M., Moriguchi K., Cho Y., Kiyokawa K., Yamamoto S., Suzuki K. (2021). Isolation and analysis of donor chromosomal genes whose deficiency is responsible for accelerating bacterial and trans-kingdom conjugations by IncP1 T4SS machinery. Front. Microbiol..

[29] Moriguchi K., Zoolkefli F.I.R.M., Abe M., Kiyokawa K., Yamamoto S., Suzuki K. (2020). Targeting antibiotic resistance genes is a better approach to block acquisition of antibiotic resistance than blocking conjugal transfer by recipient cells: A genome-wide screening in *Escherichia coli*. Front. Microbiol..

[30] Zhang P.-Y., Xu P.-P., Xia Z.-J., Wang J., Xiong J., Li Y.-Z. (2013). Combined treatment with the antibiotics kanamycin and streptomycin promotes the conjugation of *Escherichia coli*. FEMS Microbiol. Lett..

[31] Quail M.A., Guest J.R. (1995). Purification, characterization and mode of action of PdhR, the transcriptional repressor of the *pdhR–aceEF–lpd* Operon of *Escherichia coli*. Mol. Microbiol..

[32] Perham R.N. (2000). Swinging arms and swinging domains in multifunctional enzymes: Catalytic machines for multistep reactions. Annu. Rev. Biochem..

[33] Anzai T., Imamura S., Ishihama A., Shimada T. (2020). Expanded roles of pyruvate-sensing PdhR in transcription regulation of the *Escherichia coli* K-12 genome: Fatty acid catabolism and cell motility. Microb. Genom..

[34] Masai H., Arai K. (1996). DnaA- and PriA-dependent primosomes: Two distinct replication complexes for replication of *Escherichia coli* chromosome. Front. Biosci..

[35] Alalam H., Graf F.E., Palm M., Abadikhah M., Zackrisson M., Boström J., Fransson A., Hadjineophytou C., Persson L., Stenberg S. (2020). A high-throughput method for screening for genes controlling bacterial conjugation of aAntibiotic resistance. mSystems.

[36] Lee E.H., Kornberg A. (1991). Replication deficiencies in *priA* mutants of *Escherichia coli* lacking the primosomal replication n’ protein. Proc. Natl. Acad. Sci. USA.

