# Supplementary material for: Genome-Wide Survey of Donor Chromosomal Genes Involved in Trans-Kingdom Conjugation via the RP4-T4SS Machinery
Source: Microorganisms. 2025 Feb 22;13(3):488. doi: 10.3390/microorganisms13030488 (PMC11946144; doi:10.3390/microorganisms13030488)
Supplement: Supplementary file 1 [file microorganisms-13-00488-s001.zip › microorganisms-3442765-supplementary.pdf]

microorganisms

MDPI

Article

Genome-Wide Survey of Donor Chromosomal Genes Involved in Trans-Kingdom Conjugation via the RP4-T4SS Machinery

Supplementary Materials

Detailed description of the genome-wide screening process

The basic strategy of the genome-wide screening is shown in Figure S1. In the first-round screening, the relative conjugation value (RTCV) of mutant strains was distorted to a lower value compared with the median value of a control ($MTCV_{ctrl}$), which is the parental strain of Keio mutant strains and carries a plasmid with a kanamycin resistance gene (Figure S2). The median value of the log_2 converted RTCV of all mutant strains was approximately −2.5, meaning a 1/6-fold relative TKC efficiency, and 1446 (37%) strains showed less than the detectable limit (no transconjugant). We selected 1942 strains, which showed a log_2 value less than −3 relative to the median of all controls used in a single screening round or relative to the median of controls used in a single screening experiment (including approximately 180 mutants), and then performed the second-round screening.

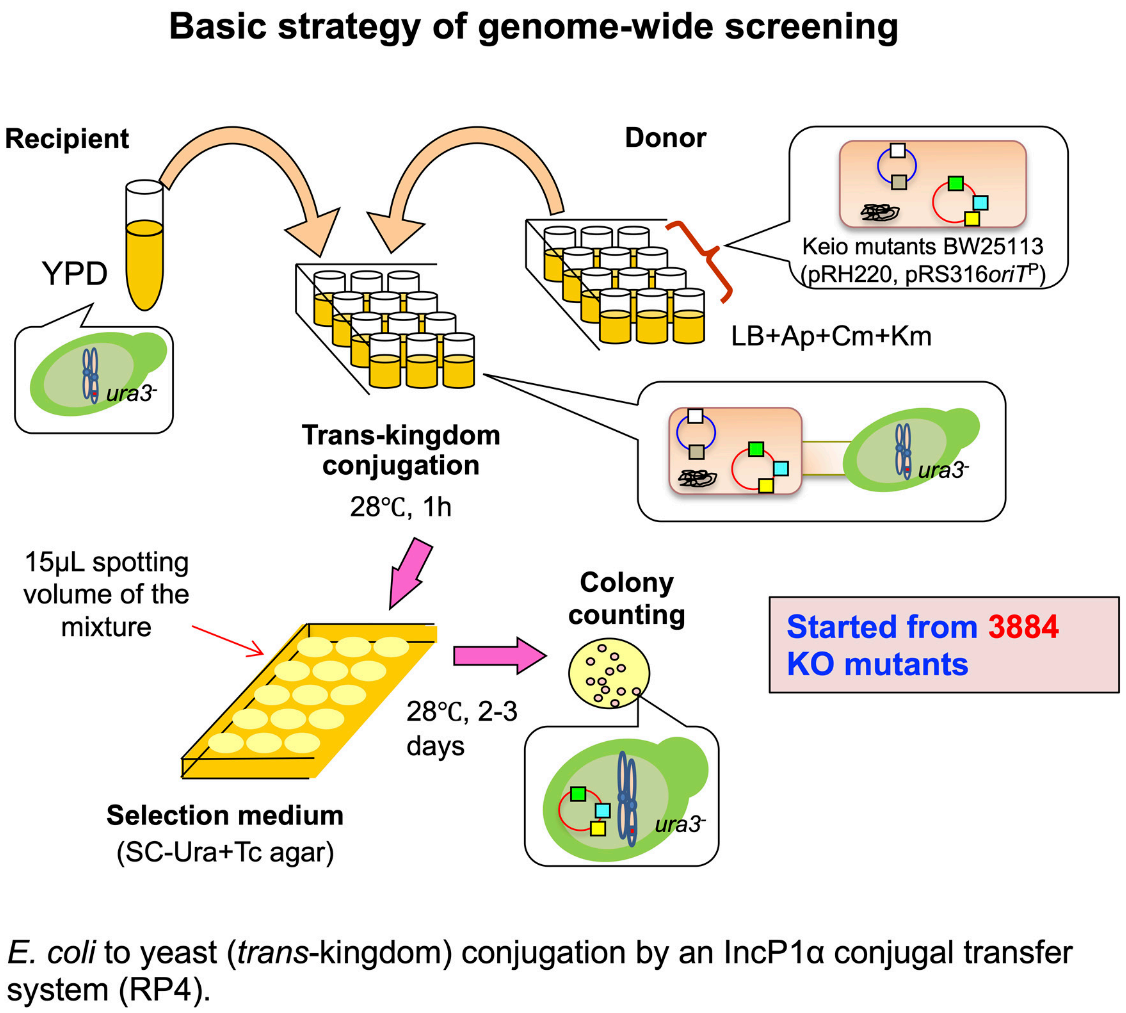

E. coli to yeast (*trans*-kingdom) conjugation by an IncP1α conjugal transfer system (RP4).

Figure S1

 https://doi.org/10.3390/microorganisms13030488

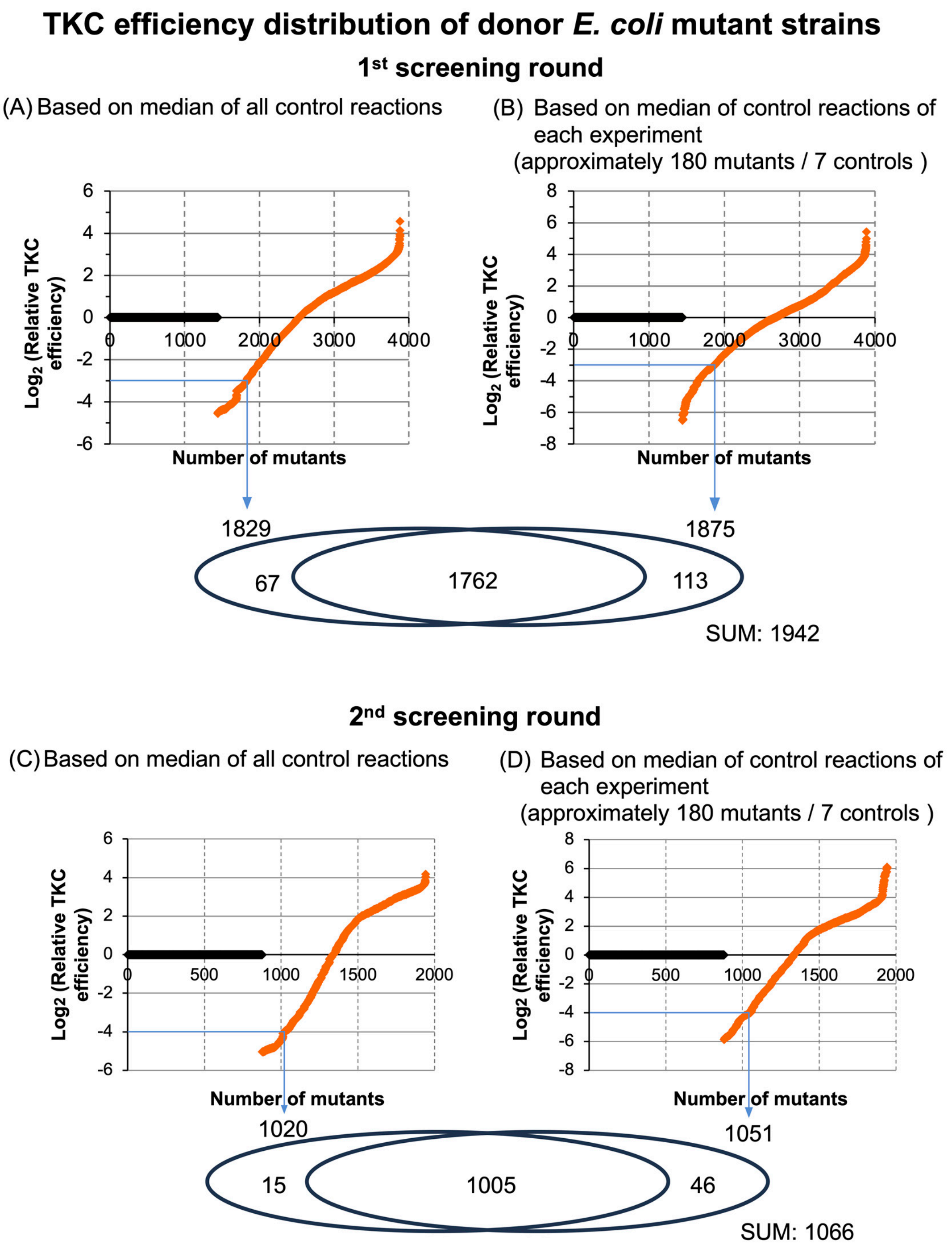

Figure S2

In the second-round screening, despite selecting low $\log_2$ value (< −3) strains in the first round, 876 (45%) strains showed a $\log_2$ value greater than or equal to −3, and 879 (45%) strains showed less than the detectable limit. This result indicated that the distortion occurred randomly, and most of the strains selected in the first round were false positives (i.e., false TKC-defective strains). Therefore, repeated rounds of screening were necessary to narrow down the true positive strains. We selected 1066 strains in the second round, which showed a $\log_2$ value less than −4.

In the third round of screening, 50 µL of the overnight donor cultures were inoculated into 100 µL of fresh medium and then cultured for 1 h at 37°C to allow for recovery. The median value of the $\log_2$ converted RTCV of the 1066 mutant strains was 0.08 (Figure S3A) and −0.10 (Figure S3B) by two respective analysis methods, showing identical levels to that of the control strain. However, this result also suggested that most of the strains were still false positives. A total of 255, 92, and 81 strains, which showed a $\log_2$ value less than −4, were selected in the third to fifth rounds of screening, respectively (Figures S3 and S4).

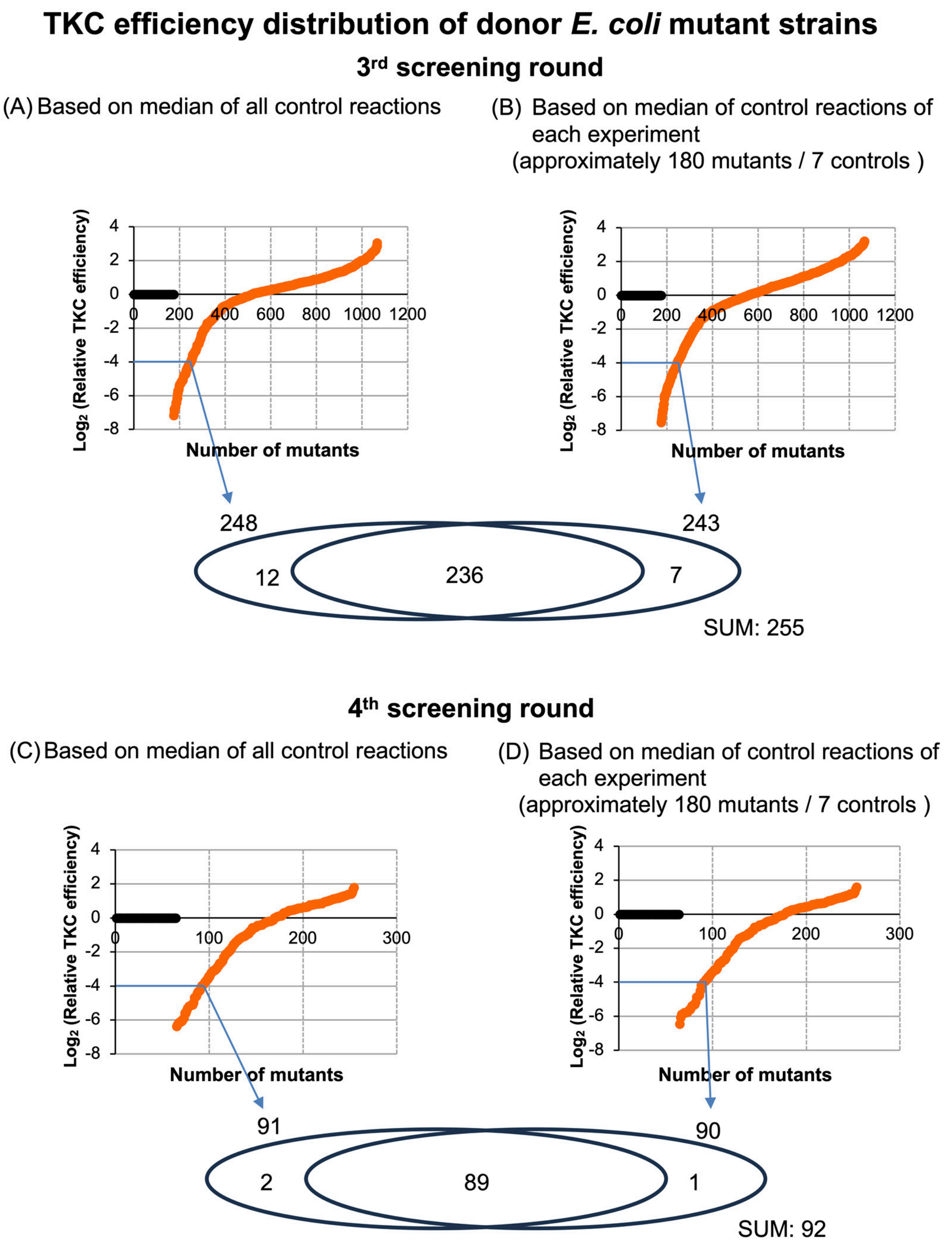

Figure S3

Because 57 (62%) strains out of 92 strains showed less than the detectable limit in the fifth round, and because strains that showed slow growth were included in that pool, we wondered whether the antibiotics used in the screening might have influenced the appearance of strains that showed a TKC efficiency less than the detectable limit. Therefore, in later steps, we excluded kanamycin from the culture medium for donor strains, which was a chromosomal selection marker but not essential for the maintenance of the donor and helper plasmids in this study. We divided the remaining 81 strains into two groups: group 1 (12 strains, mainly slow-growing strains) and group 2 (69 strains), and then performed sub-screening for the group 2 strains. Seven strains (named group 3) out of 69 strains showed a $\log_2$ value less than −4, and no strains that showed less than the detectable limit were observed (Figure S4). This result strongly suggests that a group of mutant strains had inhibited their TKC efficiency, at least in part, due to the addition of kanamycin to their cultures.

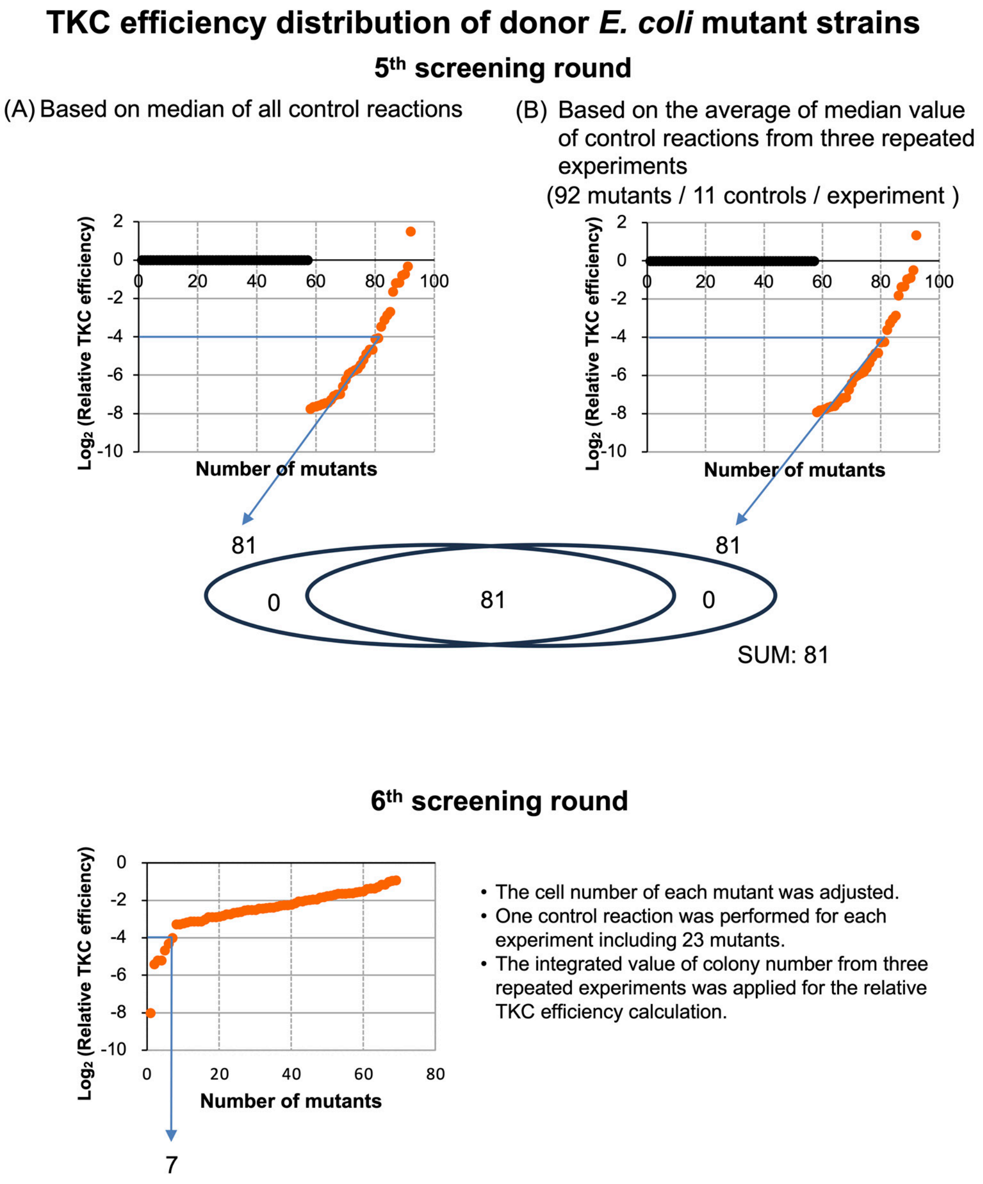

Figure S4

Because we prepared the sub-library in every screening step by inoculating screened strains from the frozen glycerol stock of the previous library, we could not eliminate the possibility that the repeated freezing of stock preparation, inoculation, and culture introduced a selection bias on TKC ability in some strains. Therefore, we measured the absolute value of TKC efficiency for the strains in group 3 using their original stock. Consistent with our worst concern, no clear TKC deficiency was observed in any of the strains in group 3. Based on this result, for the measurement of absolute TKC efficiency in the group 1 strains, the strains from their original stock were used, and we finally identified two TKC-defective mutants, Δ*aceE* and Δ*priA* (Figure 2).

TKC ability in mutants of other genes involved in pyruvate metabolism to acetyl-CoA

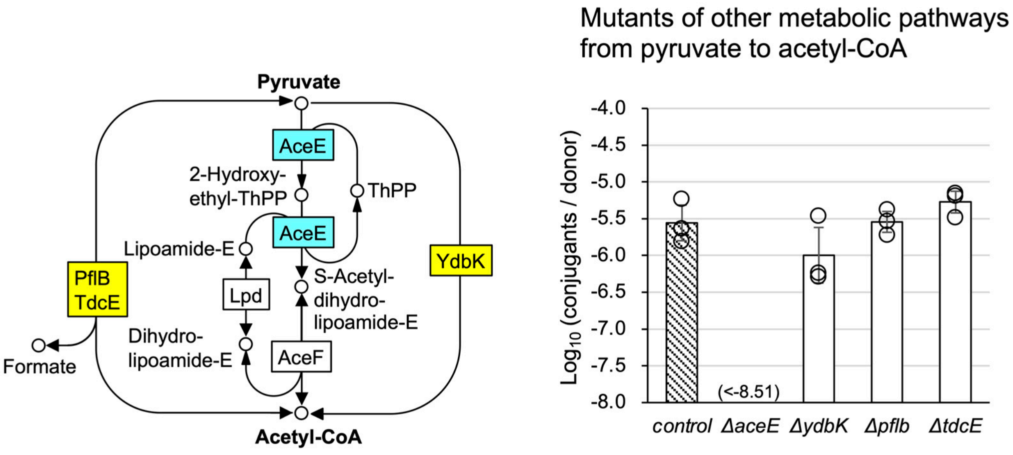

Figure S5

We further analyzed the TKC ability of knockout (KO) mutants for genes involved in the metabolic pathway from pyruvate to acetyl-CoA, excluding pyruvate dehydrogenase-related genes. The left panel illustrates the pathways associated with the analyzed mutants, as derived from the KEGG pathway map (https://www.kegg.jp/pathway/map=eco00620&keyword=aceE). The *ydbK* gene encodes pyruvate-ferredoxin/flavodoxin oxidoreductase, while *pflB* and *tdcE* encode formate C-acetyltransferase. All analyses were performed in triplicate, and data are presented as the mean ± standard deviation (SD). No significant differences in TKC ability were observed between the control strain (parental strain) and the respective KO mutants (*ΔydbK*, *ΔpflB*, and *ΔtdcE*) based on a two-tailed t-test.
